# Micro and macropolitical determinants for non-vaccination against COVID-19 in pregnant women in Belo Horizonte

**DOI:** 10.1590/0034-7167-2023-0235

**Published:** 2025-01-10

**Authors:** Marina Stuart Marques, Thales Philipe Rodrigues da Silva, Ana Paula Vieira Faria, Nágela Cristine Pinheiro Santos, Janaína Fonseca Almeida Souza, Marla Ariana Silva, Patrícia Feliciano Pereira, Fernanda Penido Matozinhos

**Affiliations:** I Universidade Federal de Minas Gerais. Belo Horizonte, Minas Gerais, Brazil; II Universidade Federal de São João del-Rei. Divinópolis, Minas Gerais, Brazil; III Universidade Federal de Viçosa. Viçosa, Minas Gerais, Brazil

**Keywords:** Vaccination, Pregnant Women, SARS-CoV-2, COVID-19, Nursing, Vacunación, Mujeres Embarazadas, SARS-CoV-2, COVID-19, Enfermería

## Abstract

**Objective:**

To analyze the determinants for non-vaccination against COVID-19 in pregnant women in Belo Horizonte, Minas Gerais, Brazil.

**Methods:**

An epidemiological study with a cross-sectional design was conducted using data from the project titled “Childbirth and Breastfeeding in Children of Mothers Infected by SARS-CoV-2,” developed during the pandemic in the city of Belo Horizonte, Minas Gerais, Brazil.

**Results:**

The study sample consisted of 360 pregnant women, of whom 77.89% received the COVID-19 vaccine. External, social, and institutional determinants can influence lower adherence to COVID-19 vaccination, especially the absence of employment during pregnancy, difficult access to prenatal consultations, and a compromised or deficient support network.

**Conclusions:**

In light of this scenario, greater encouragement for health education is necessary, especially during prenatal care, resulting in lower rates of morbidity and mortality and favorable perinatal outcomes.

## RESUMEN

**Objetivo:**

Analizar los determinantes para la no vacunación contra la COVID-19 en gestantes de Belo Horizonte, Minas Gerais, Brasil.

**Métodos:**

Estudio epidemiológico, con diseño transversal, realizado con datos del proyecto titulado “Parto y Lactancia Materna en Hijos de Madres Infectadas por SARS-CoV-2”, desarrollado durante la pandemia en la ciudad de Belo Horizonte, Minas Gerais, Brasil.

**Resultados:**

La muestra de este estudio estuvo compuesta por 360 gestantes, de las cuales el 77,89% recibió la vacuna contra la COVID-19. Determinantes externos, sociales e institucionales pueden influir en la menor adhesión a la vacunación contra la COVID-19, especialmente: ausencia de trabajo durante la gestación, acceso dificultado a consultas prenatales y red de apoyo comprometida o deficiente.

**Conclusiones:**

Ante este escenario, es necesario un mayor incentivo a la educación en salud, sobre todo en el momento de la asistencia prenatal, resultando en menores tasas de morbimortalidad y resultados perinatales favorables.

## INTRODUCTION

In December 2019, in a province in China, an outbreak of a virus later known worldwide as the novel coronavirus, SARS-CoV-2, emerged, causing the disease known as coronavirus disease 2019 (COVID-19)^(1)^. In response to this scenario, the World Health Organization (WHO) declared a public health emergency of international concern in March 2020^(2)^. Considering the high transmissibility of the COVID-19 virus, social distancing became an essential measure to reduce interactions between infected individuals or those who were asymptomatic within a community. Thus, social distancing helps decrease the likelihood of contagion^(3)^.

Given the rapid spread of the virus and the high demand for health services, a global quest for the development of a vaccine against this virus began. In Brazil, given the epidemiological emergency caused by COVID-19, the emergency use of vaccines against COVID-19 was temporarily authorized in an experimental capacity to combat the pandemic resulting from the dissemination of SARS-CoV-2^(4)^.

Among the immunobiologicals approved for emergency use worldwide, the National Health Surveillance Agency (ANVISA) approved four different types of vaccines: AstraZeneca, Sinovac/CoronaVac, Pfizer, and Janssen^(5)^. In the context of pregnant women, it is known that immunized pregnant women who were infected with COVID-19 presented mild symptoms. Among those requiring ventilatory support and mechanical ventilation, most were not vaccinated, revealing a positive impact of vaccination in preventing the disease, especially in pregnant women^(4)^.

In this context, the National Immunization Program (PNI) developed and published the National COVID-19 Vaccination Operationalization Plan (PNO), which defined the priority groups for COVID-19 vaccination. Consequently, on March 15, 2021, the Ministry of Health (MS) included pregnant women with comorbidities as a priority group for receiving the COVID-19 vaccine. On April 27 of the same year, pregnant and postpartum women were included in the vaccination campaign^(6)^.

Among the at-risk groups are pregnant women, as they are more susceptible to infections, such as viral ones, compared to non-pregnant women^(7)^. It is noted that pregnant women infected with COVID-19 have a higher predisposition for negative outcomes, such as a high rate of intensive care unit (ICU) admission, the need for supplemental oxygen, and a higher level of mortality compared to non-pregnant women^(4)^.

Under this perspective, after the start of vaccination for the group of pregnant and postpartum women, on May 7, 2021, the manufacturer of the Oxford/AstraZeneca/Fiocruz vaccine notified ANVISA of a suspected serious adverse event of hemorrhagic stroke with thrombocytopenia in a pregnant woman, resulting in fetal death and subsequent maternal death^(5)^. In light of this scenario, the Ministry of Health (MS) approved only the Pfizer (mRNA vaccine) and CoronaVac (inactivated virus vaccine) for use in pregnant women^(6)^.

Regarding the number of deaths, according to a bulletin provided by the Obstetric Observatory, COVID-19 caused the deaths of 2,053 pregnant and postpartum women in the country, with 2021 accounting for the majority of cases (74%). Specifically, there were 462 maternal deaths in 2020, 1,519 deaths in 2021, 72 deaths in 2022, and, so far, no deaths in 2023^(8)^.

According to the Obstetric Observatory COVID-19 Vaccination (2023), 2,657,415 doses of the COVID-19 vaccine were administered to pregnant and postpartum women, with 38.84% having received the first dose and 35.49% having received the second dose or a single dose^(8)^. Although pregnant and lactating women were not included as participants in the clinical studies and trials for vaccine testing, professional societies such as the American College of Obstetricians and Gynecologists and the Society for Maternal-Fetal Medicine recommend vaccination for this group^(9)^. Vaccination during pregnancy is an important strategy as it allows the mother to develop active immunity against serious infectious diseases and ensures the protection of the newborn against illnesses with high morbidity and mortality rates. However, despite the benefits of vaccination outweighing the potential risks of not vaccinating, some people have concerns regarding the safety of the mRNA vaccine, as it is a new vaccine^(10)^.

This study is relevant as it brings to light information that can guide future research on the subject, directing the daily practices of multidisciplinary teams and positively influencing these professionals. Furthermore, it can be used as a source of information for health education and guidance provided by nursing teams to the target audience and aims to contribute positively and additively to the data related to the obstetric outcomes of SARS-CoV-2 in vaccinated and unvaccinated postpartum women.

The hypothesis of this study is that, in addition to individual factors or decisions, other external, social, or institutional determinants (micro and macro-political), such as those inherent to health services or support networks, can influence non-adherence to COVID-19 vaccination in pregnant women. Finally, the guiding research question is: What are the determinants associated with non-vaccination against COVID-19 in pregnant women?

## OBJECTIVE

To analyze the determinants for non-vaccination against COVID-19 in pregnant women in Belo Horizonte, Minas Gerais, Brazil.

## METHODS

### Ethical Aspects

The study was approved by the Research Ethics Committee (CEP) of the Federal University of Minas Gerais (UFMG). Informed consent was obtained from all postpartum women involved in the study through telephone collection and recording of the call.

### Study Design, Period, and Location

This is an epidemiological study with a cross-sectional design, guided by the STROBE (Strengthening the Reporting of Observational Studies in Epidemiology) tool, developed from data from the project titled “Childbirth and Breastfeeding in Children of Mothers Infected by SARS-CoV-2.” The research on the medical records of the parturients was conducted in 2020 in three reference maternity hospitals in the city of Belo Horizonte, Minas Gerais, Brazil. Subsequently, telephone contacts were made with the postpartum women from 2020 to 2022.

One of the hospitals serves a population exceeding 400,000 inhabitants from the city of Belo Horizonte and other municipalities through the Central Regulator of the Belo Horizonte Municipal Health Department. This institution is a philanthropic hospital located in the Northern Sanitary District, highlighted by the Ministry of Health as a model of evidence-based care in humanized practices for newborns and for adopting the Stork Network as a public health policy, providing a total of 951 assisted deliveries per month^(11)^.

The second hospital assists approximately 250 deliveries per month and is also a relevant site for urgent and emergency care and maternal and child health. It is a philanthropic institution managed by the Federal University of Minas Gerais^(12)^. Finally, the third is a public regional hospital that offers urgent care services, comprehensive care for patients with respiratory conditions and complex pathologies, and global health care for children and adolescents, among others. However, it is notably recognized in the field of gynecology and obstetrics for the care provided to women^(13)^.

Regarding the sample size calculation, a cohort study design was used, considering a ratio of nine pregnant women for the control group (pregnant women not exposed to COVID-19) for each pregnant woman in the case group (woman exposed to COVID-19), due to the high infection rate of 10% during the epidemic period^(14)^. To achieve a 95% confidence interval and 80% power for the sample, an Odds Ratio of 1.5 was estimated. Based on these parameters, a final sample of 2,267 parturients was obtained, with the division of pregnant women by maternity considered based on the proportion of the total number of births in each defined institution. The pregnant women were contacted at various times, and the telephone contact was made with at least 5 attempts by trained researchers. In cases of refusals or unsuccessful attempts, the postpartum woman was excluded/ substituted. Thus, the data collection follow-up consisted of 360 pregnant women who responded to the telephone contact during the data collection follow-up.

### Sample, Inclusion, and Exclusion Criteria

For sample selection, the period with the highest incidence of COVID-19 cases was chosen for analysis of medical records, specifically May, June, and July 2020. From these records, those meeting the inclusion criteria were selected, considering eligible all single pregnancies with hospital deliveries, where newborns (NB) were conceived at 22 weeks of gestation or more; live NBs weighing more than 500 grams at birth; excluding women under 18 years old. Finally, through random selection, the parturients were chosen from the birth and registration book, and subsequently, their medical records were evaluated in the reference hospitals.

### Study Protocol

Data collection was conducted by trained professionals through the analysis of selected medical records from each hospital institution in the study. A structured questionnaire adapted from the research “Childbirth and Breastfeeding in Children of Mothers Infected by SARS-CoV-2” was used as the data collection instrument to analyze the clinical-obstetric history, labor and delivery assistance in the observed institutions, birth methods, maternal clinical changes throughout hospitalization, breastfeeding, and COVID-19 infection. The confirmation of SARS-CoV-2 infection in women was verified from hospital records. In symptomatic women, the confirmatory test performed at the institution and its respective result were sought. In cases where the test was not performed, pregnant women presenting symptoms suggestive of the infection at the time of admission were considered suspected cases. The dependent variable was non-vaccination against COVID-19 in pregnant women in Belo Horizonte. The independent variables were divided into sociodemographic variables (age, education, income, marital status, skin color, and occupation during pregnancy), obstetric history variables (parity, abortion history, and number of prenatal consultations), micropolitical variables (difficult access to prenatal consultations and receiving guidance against COVID-19 during prenatal care), and macropolitical variables (receiving the COVID-19 vaccine).

### Data Analysis and Statistics

The data obtained were stored in a spreadsheet using Microsoft Office Excel® 2010. Subsequently, they were analyzed using the Statistical Software for Professionals (Stata), version 17.0, and presented through absolute and relative frequencies. The variables were controlled by age, parity, abortion history, education, number of prenatal consultations, income, marital status, and self-reported skin color. Initially, the categorical data were presented using absolute frequency, relative frequency, and their respective confidence intervals (CI 95%). Poisson regression was also performed to estimate the parameters of interest: crude and adjusted factors associated with non-vaccination against COVID-19 in postpartum women in Belo Horizonte. The construction of the multivariate regression model followed the backward method, including all variables of interest at a significance level of less than 20% in the bivariate analysis or based on theoretical criteria^(15)^. The Hosmer-Lemeshow test was used to verify the fit of the final model. The crude and adjusted prevalence ratios were presented, and the 95% confidence intervals (CI95%) were calculated, considering a significance level of 5% in all analytical procedures.

## RESULTS

The sample of this study consisted of 360 pregnant women, of whom 77.89% received the COVID-19 vaccine. Regarding the sociodemographic profile, 59.36% of the pregnant women were under 30 years old; 73.06% had completed higher education or high school; 50% had an income of up to one minimum wage or more or had no income; 60.83% were married or in a stable union; 84.44% self-identified as Black, Brown, Asian, or Indigenous; and 66.22% reported working during pregnancy.

Regarding obstetric history, 88.61% were multiparous; 72.76% had no history of abortion; 84.05% reported having more than six prenatal consultations; 67.78% reported no difficulty in accessing prenatal consultations, and 88.06% had a postpartum support network. Finally, regarding COVID-19 vaccination, 65.03% received guidance on COVID-19; 77.89% of them were vaccinated, with 45.92% immunized with the Pfizer vaccine (Table 1).

**Table 1:** Demographic, socioeconomic, and obstetric profile of the sample of pregnant women. Belo Horizonte, Minas Gerais, Brazil, 2020-2022 (N=360)

	n (%)	95%CI
Sociodemographic profile		
Age		
30 years old or younger	149 (59.36)	53.12-65.30
Older than 30 years	102 (40.64)	34.69-46.87
Education		
Higher education/high school	263 (73.06)	68.21 -77.40
Elementary/primary/illiterate	97 (26.94)	22.59 – 31.78
Income		
1 minimum wage or more	174 (50.0)	44.74-55.25
No income/up to 1 minimum wage	174 (50.0)	44.74-55.25
Marital status		
Married/stable union	219 (60.83)	55.66-65.76
Single/widowed/divorced	141 (39.17)	34.23-44.33
Skin color		
White	56 (15.56)	12.15-19.69
Black/brown/asian/indigenous	304 (84.44)	80.30-87.84
Worked during pregnancy		
Yes	224 (66.22)	57.07-67.10
No	136 (37.78)	32.89-42.92
Obstetric history		
Parity		
Primiparous	41 (11.39)	08.48-15.12
Multiparous	319 (88.61)	84.87-91.51
Abortion history		
No	235 (72.76)	67.61-77.35
Yes	88 (27.24)	22.64-32.38
Number of Prenatal Consultations		
6 or more	216 (84.05)	79.01- 88.05
Fewer than 6	41 (15.95)	11.94-20.98
Difficulty accessing prenatal consultations		
No	244 (67.78)	62.74-72.42
Yes	116 (32.22)	27.57-37.25
Postpartum support network		
Yes	317 (88.06)	84.25-91.03
No	43 (11.94)	08.96-15.74
Vacinação		
Received guidance on COVID-19 during Prenatal Care		
Yes	186 (65.03)	59.29-70.37
No	100 (34.97)	29.62-40.70
Received the COVID-19 vaccine		
Yes	236 (77.89)	72.83-82.22
No	67 (22.11)	17.77-27.16
Which vaccine?		
CoronaVac	61 (26.18)	20.90-32.24
Oxford-Astrazeneza	63 (27.04)	21.68-33.14
Pfizer	107 (45.92)	39.57-52.40
Janssen	02 (00.86)	00.21-03.39

*Notes: n = Sample number; CI95% = 95% confidence interval; The totals of the variables (n) may vary due to data loss in each of them.*

Through the analysis of prevalence ratios and the bivariate analysis of factors associated with non-vaccination against COVID-19 among the pregnant women in the study, it was observed that the following were predominant among non-vaccinated pregnant women: age under 30 years (68.39%), multiparous (22.47%), elementary or primary education or illiterate (25.86%), no history of abortion (24.10%), more than six prenatal consultations (18.09%), report of not receiving guidance on COVID-19 during prenatal care (20.69%), no income or income up to one minimum wage (27.34%), single/widowed/divorced (25.86%), self-identified as Black, Brown, Asian, or Indigenous (22.44%), did not work during pregnancy (29.31%), had difficulty accessing prenatal consultations (23.71%), and reported not having a support network (38.24%).

A statistically significant difference was observed (p<0.018) regarding whether the woman worked during the pandemic, as well as concerning the support network (p<0.010). In the bivariate analysis, an association was found between non-vaccination and paid work during prenatal care and the support network, showing a statistically significant difference (Table 2).

**Table 2:** Prevalence analysis of non-vaccination and bivariate analysis of factors associated with non-vaccination against COVID-19 in pregnant women. Belo Horizonte, Minas Gerais, Brazil, 2020-2022

	Not Vaccinated n=67 n (%)	Unadjusted Analysis	
		PR1 (95%CI)	*p* value
Age			0.188
30 years old or younger	31 (68.39)		
Older than 30 years	14 (31.11)	0.682 (0.387-1.204)	
Parity			0.686
Primiparous	7 (19.44)		
Multiparous	60 (22.47)	1.151 (0.572 – 2.332)	
Education			0.777
Higher education/high school	37 (19.79)	1	
Elementary/primary/illiterate	30 (25.86)	0.931 (0.571-1.519)	
Abortion history			0.226
No	47 (24.10)	1	
Yes	13 (17.11)	0.709 (0.407-1.236)	
Number of Prenatal Consultations			0.895
6 or more	34 (18.09)	1	
Fewer than 6	6 (17.14)	0.947 (0.429-2.090)	
Received guidance on COVID-19 during Prenatal Care			0.955
Yes	32 (20.38)	1	
No	18 (20.69)	1.012 (0.667-1.534)	
Income			0.056
1 minimum wage or more	28 (17.95)	1	
No income/up to 1 minimum wage	38 (27.34)	1.523 (0.998-2.346)	
Marital status			0.215
Married/stable union	37 (19.79)	1	
Single/widowed/divorced	30 (25.86)	1.307 (0.856-1.995)	
Skin color			0.881
White	10 (21.28)	1	
Black/brown/asian/indigenous	57 (22.44)	1.04 (0.899-1.131)	
Worked during pregnancy			0.018
Yes	33 (17.65)	1	
No	34 (29.31)	1.660 (1.091-1.527)	
Difficulty accessing prenatal consultations			0.644
No	44 (21.36)	1	
Yes	23 (23.71)	1.110 (0.712-1.729)	
Support network			0.010
Yes	54 (20.07)	1	
No	13 (38.24)	1.904 (1.166-3.109)	

*Notes: n = Sample number; PR = Prevalence ratio; CI95% = 95% confidence interval; The totals of the variables (n) may vary due to data loss in each of them.*

In the adjusted analyses, it was observed that, after adjusting for other variables, the condition of not working during pregnancy increased the prevalence ratio of a woman not receiving the COVID-19 vaccine by an average of 2.15 times compared to those who worked during pregnancy. Having difficulty accessing health services during prenatal care increased the prevalence ratio of a woman not receiving the COVID-19 vaccine by an average of 1.93 times compared to women who did not have difficulty accessing health services. Not having a support network increased the prevalence ratio of a woman not receiving the COVID-19 vaccine by an average of 2.47 times compared to those who had some type of support network during the postpartum period (Table 3).

**Table 3:** Prevalence analyses of non-vaccination and bivariate analyses of factors associated with non-vaccination against COVID-19 in pregnant women. Belo Horizonte, Minas Gerais, Brazil, 2020-2022

	Unadjusted Analysis^2^	
	PR^2^ (95%CI)	*p* value
Worked during pregnancy		
Yes	1	
No	2.15 (1.759-3.957)	0.013
Difficulty accessing prenatal consultations		
No	1	
Yes	1.93 (1.044-3.576)	0.036
Support network		
Yes	1	
No	2.47 (1.322-4.621)	0.005

*Notes: PR = Prevalence Ratio; CI95% = 95% Confidence Interval.*

## DISCUSSION

In this study, it was observed that 77.89% of pregnant women were vaccinated against COVID-19, revealing that a significant portion of this group did not adhere to vaccination. In addition to the sociodemographic and obstetric history factors analyzed, other external or social factors, such as those inherent to health services or support networks, as well as whether or not the women worked during pregnancy, can influence postpartum women not to get vaccinated. A study conducted in Pakistan in 2020 reported that factors such as demographic and financial characteristics interfere in the process of vaccine hesitancy against COVID-19^(16)^.

Regarding vaccination coverage, there is an important relationship between obstetric inequalities and vaccination. Recognizing the social determinants of health requires considering social, economic, cultural, and environmental issues that affect an individual’s health, influencing existing social inequalities and, consequently, reducing vaccination coverage in certain population groups^(17)^.

The unequal conditions in which certain people find themselves correspond to health inequities, which refer to specific contexts where there is unequal access, or even lack of access, to fundamental human rights that ensure a minimum necessary level to provide a dignified and fair life^(18)^.

A study conducted in Italy between 2005 and 2010, using data obtained from the Standard Live Birth Certificate administrative source, demonstrated a dependency relationship between the use of prenatal (PN) services and sociodemographic aspects^(19)^. It is known that women with low education levels, who are single, or unemployed have a higher chance of inadequately utilizing prenatal services. Similarly, “dysfunctional” family contexts, such as single-parent families or unhealthy relationships between the paternal figure and the child, considerably interfere with prenatal consultations^(19)^.

In the present study, women without partners had less access to prenatal consultations and later initiation of care. In contrast, there is a strong association between marital status and adequate use of prenatal care, as married women or those living with their partners find it easier to access the service, as they have a support network to assist them in this process, including in immunization actions^(20)^.

A recent study with pregnant women in Sweden showed that many of them were deprived of information from healthcare professionals during the pandemic, including information about immunization efforts^(21)^. An Australian cross-sectional study stated that only one-third of the pregnant women surveyed received quality prenatal education^(21)^.

Adequate prenatal care is an essential tool for promoting women’s health during the perinatal period, as practices carried out during this process are associated with better perinatal outcomes. In this context, healthcare professionals, by establishing a relationship with the pregnant woman, can guide her on pregnancy care, such as the importance of COVID-19 vaccination for her and her baby’s protection.

This study found that socioeconomic factors and access to healthcare services are determinants for higher vaccination coverage during pregnancy. It was also identified that paid work and the number of prenatal consultations were associated with a lower proportion of non-vaccination in pregnant women. Therefore, employment is an important factor for adequate prenatal care, as similar studies have found a connection between women working and the early initiation of prenatal consultations, which consequently leads to a higher probability of maternal immunization against COVID-19 and other diseases^(22)^.

Thus, a woman working can positively influence access to prenatal care and health maintenance due to greater access to information and a higher level of education. The care provided during the prenatal period is directly related to pregnant women’s vaccination, being a significant factor that directly affects vaccination coverage, as it provides the pregnant woman with significant knowledge about the protection conferred by immunobiologicals^(22)^.

Employed pregnant women (79.4%) had an acceptance rate of the COVID-19 vaccine 2.44 times higher than those who did not work (52%). Furthermore, employers can encourage or even require vaccination as part of their working conditions, especially in certain sectors, and thus employed pregnant women might have higher adherence to vaccination^(23)^. Employed mothers may use prenatal services more frequently because information about pregnancy risks is widely available in the workplace^(24)^.

### Study Limitations

Finally, some limitations of this work should be noted, such as the fact that, among the 67 unvaccinated pregnant women, we did not find other associations for non-vaccination. This means that there may be additional factors to be investigated, beyond individual willingness or the actual desire not to get vaccinated. Nevertheless, the rigorous methodology used in this study and the scarcity of national studies on this topic are emphasized, to the best of our knowledge.

### Contributions to the Field of Nursing

This study contributes to the field of nursing as it provides information that can guide future research on the topic, directing the daily practices of multidisciplinary teams and positively influencing these professionals. Furthermore, it can be used as a source of information for health education and guidance provided by nursing teams to the target audience, contributing positively and additively to the data related to the obstetric outcomes of SARS-CoV-2 in vaccinated and unvaccinated postpartum women. Additionally, it can help increase vaccination coverage by making the vaccine more accessible due to the relevant information.

## CONCLUSIONS

From this work, it is concluded that external, social, and institutional determinants can influence lower adherence to COVID-19 vaccination, particularly: absence of work during pregnancy, difficult access to prenatal consultations, and a compromised or deficient support network. Given the findings presented here, this study reinforces and advances the understanding of the benefits of COVID-19 vaccination for the female population.

In light of this scenario, greater encouragement for health education is necessary, especially during prenatal care, so that healthcare professionals can emphasize the importance of vaccination during pregnancy and postpartum as a protective factor, resulting in lower morbidity and mortality rates and, consequently, favorable perinatal outcomes. It is hoped that this study can contribute to the adherence of postpartum women to COVID-19 vaccination, aiming to mitigate the negative effects of the pandemic by preventing diseases such as coronavirus.
