# Supplementary material for: Micro and macropolitical determinants for non-vaccination against COVID-19 in pregnant women in Belo Horizonte
Source: Rev Bras Enferm. 2025 Jan 10;77(Suppl 1):e20230235. doi: 10.1590/0034-7167-2023-0235 (PMC11726902; doi:10.1590/0034-7167-2023-0235)
Supplement: Translation

# Translation: Determinantes micro e macropolíticos para a não-vacinação contra a COVID-19 em gestantes de Belo Horizonte

**DOI:** 10.1590/0034-7167-2023-0235pt

**Authors:** Marina Stuart Marques, Thales Philipe Rodrigues da Silva, Ana Paula Vieira Faria, Nágela Cristine Pinheiro Santos, Janaína Fonseca Almeida Souza, Marla Ariana Silva, Patrícia Feliciano Pereira, Fernanda Penido Matozinhos

**Affiliations:** I Universidade Federal de Minas Gerais. Belo Horizonte, Minas Gerais, Brasil; II Universidade Federal de São João del-Rei. Divinópolis, Minas Gerais, Brasil; III Universidade Federal de Viçosa. Viçosa, Minas Gerais, Brasil

**Keywords:** Vacinação, Gestantes, SARS-CoV-2, COVID-19, Enfermagem

## Abstract

**Objetivo:**

Analisar os determinantes para a não-vacinação contra a COVID-19 em gestantes de Belo Horizonte, Minas Gerais, Brasil.

**Métodos:**

Estudo epidemiológico, com delineamento transversal, realizado com dados do projeto intitulado “Parto e Aleitamento Materno em Filhos de Mães Infectadas por SARS-CoV-2”, desenvolvido durante a pandemia na cidade de Belo Horizonte, Minas Gerais, Brasil.

**Resultados:**

A amostra deste estudo foi composta por 360 gestantes, das quais 77,89% receberam a vacina contra a COVID-19. Determinantes externos, sociais e institucionais podem influenciar na menor adesão à vacinação contra a COVID-19, especialmente: ausência de trabalho durante a gestação, acesso dificultado a consultas de pré-natal e rede de apoio comprometida ou deficiente.

**Conclusões:**

Diante desse cenário, é necessário um maior incentivo à educação em saúde, sobretudo no momento da assistência ao pré-natal, resultando em menores taxas de morbimortalidade e desfechos perinatais favoráveis.

## INTRODUÇÃO

Em dezembro de 2019, em uma província da China, surgiu um surto de um vírus que mais tarde receberia notoriedade mundial, denominado novo coronavírus, SARS-CoV-2, o qual causa a doença denominada *coronavirus disease* 2019 (COVID-19)^(1)^. Em razão desse cenário, a Organização Mundial da Saúde (OMS) declarou, em março de 2020, emergência de saúde pública de importância internacional^(2)^. Considerando a alta transmissibilidade do vírus da COVID-19, o distanciamento social tornou-se uma medida essencial para reduzir as interações entre as pessoas de uma comunidade infectadas ou que ainda estavam assintomáticas. Assim, o distanciamento social permite diminuir as possibilidades de contágio^(3)^.

Diante da elevada propagação do vírus e da alta demanda dos serviços de saúde, iniciou-se uma busca mundial incessante pelo desenvolvimento de uma vacina contra esse vírus. No Brasil, diante do quadro de emergência epidemiológica decorrente da COVID-19, foi autorizado temporariamente o uso emergencial, em caráter experimental, de vacinas contra a COVID-19 para o enfrentamento da pandemia oriunda da disseminação do SARS-CoV-2^(4)^.

Dos imunobiológicos aprovados para uso emergencial mundialmente, a Agência Nacional de Vigilância Sanitária (ANVISA) aprovou quatro tipos diferentes de vacinas: AstraZeneca, Sinovac/CoronaVac, Pfizer e Janssen^(5)^. No contexto das gestantes, sabe-se que gestantes imunizadas e que foram infectadas pela COVID-19 apresentaram sintomas brandos, e entre aquelas que necessitavam de suporte ventilatório e ventilação mecânica, a maioria não estava vacinada, revelando, portanto, um impacto positivo da vacinação para a prevenção da doença, especialmente nas gestantes^(4)^. Nesse contexto, o Programa Nacional de Imunizações (PNI) elaborou e publicou o Plano Nacional de Operacionalização da Vacinação contra a COVID-19 (PNO), em que foram definidos os grupos prioritários para vacinação contra a COVID-19. Diante disso, o Ministério da Saúde (MS) incluiu, no dia 15 de março de 2021, as gestantes com comorbidades como grupo prioritário para receber a vacina contra a COVID-19. Em 27 de abril do mesmo ano, grávidas e puérperas foram contempladas com a vacinação^(6)^.

Entre os grupos de risco encontra-se o de mulheres grávidas, tendo em vista que são mais suscetíveis a infecções, como as virais, em comparação com mulheres não gestantes^(7)^. Percebe-se que as gestantes infectadas pela doença apresentam maior predisposição para desfechos negativos, como alta taxa de internação em Unidade de Terapia Intensiva (UTI), necessidade de oxigênio suplementar e elevado nível de mortalidade, em comparação com mulheres não grávidas^(4)^.

Sob essa perspectiva, após o início da vacinação do grupo de gestantes e puérperas, foi notificada à ANVISA, no dia 7 de maio de 2021, pelo próprio fabricante da vacina Oxford/AstraZeneca/ Fiocruz, a suspeita de evento adverso grave de acidente vascular cerebral hemorrágico com plaquetopenia ocorrido em gestante, com confirmação de óbito fetal e posterior morte materna^(5)^. Em virtude desse cenário, o MS aprovou apenas os imunizantes Pfizer (vacina de mRNA) e CoronaVac (vacina de vírus inativado) para serem aplicados no grupo das grávidas^(6)^.

Em relação ao número de óbitos, conforme boletim disponibilizado pelo Observatório Obstétrico, a COVID-19 ocasionou a morte de 2.053 gestantes e puérperas no país, sendo que o ano de 2021 concentrou a maioria dos casos (74%). Diante disso, foram 462 óbitos maternos em 2020, 1.519 óbitos em 2021, 72 óbitos em 2022 e, até o momento, nenhum óbito em 2023^(8)^.

De acordo com o Observatório Obstétrico COVID-19 Vacinação (2023), foram administradas 2.657.415 doses de vacina contra a COVID-19 em gestantes e puérperas, sendo que, desse total, 38,84% tomaram a primeira dose, enquanto 35,49% receberam a segunda dose ou dose única^(8)^.

Embora gestantes e lactantes não tenham sido incluídas como participantes dos estudos e ensaios clínicos para teste das vacinas, sociedades profissionais como o *American College of Obstetricians and Gynecologists* e a *Society for Maternal-Fetal Medicine* recomendam a vacinação para esse grupo^(9)^. A vacinação durante a gravidez configura-se como uma importante estratégia, pois permite a imunidade ativa da mãe contra doenças infecciosas graves, bem como garante a proteção do neonato diante de enfermidades que apresentam altas taxas de morbimortalidade. Contudo, apesar de os benefícios de se vacinar superarem os potenciais riscos da não vacinação, algumas pessoas apresentam receio em relação à segurança da vacina de mRNA, por ser uma vacina nova^(10)^.

O presente estudo é relevante por trazer à tona informações que podem nortear estudos futuros acerca da temática, direcionando os profissionais da equipe multidisciplinar no seu cotidiano e influindo de forma positiva na prática desses profissionais. Ademais, pode ser utilizado como fonte de informação para educação e orientação em saúde ao público-alvo e almeja contribuir de forma positiva e aditiva nos dados relacionados aos desfechos obstétricos da SARS-CoV-2 em puérperas vacinadas e não vacinadas.

Tem-se como hipótese deste estudo que, além dos fatores ou decisões individuais, outros determinantes externos, sociais ou institucionais (micro e macropolíticos), como os inerentes aos serviços de saúde ou rede de apoio, podem influenciar na não adesão à vacinação contra a COVID-19 em gestantes. Por fim, assume-se como pergunta norteadora de pesquisa: quais são os determinantes associados à não-vacinação contra a COVID-19 em gestantes?

## OBJETIVO

Analisar os determinantes para a não-vacinação contra a COVID-19 em gestantes de Belo Horizonte, Minas Gerais, Brasil.

## MÉTODOS

### Aspectos Éticos

O estudo foi aprovado pelo Comitê de Ética em Pesquisa (CEP) da Universidade Federal de Minas Gerais (UFMG). O Consentimento Livre e Esclarecido foi obtido de todas as puérperas envolvidas no estudo por meio de coleta telefônica e gravação da ligação.

### Desenho, período e local do estudo

Trata-se de um estudo epidemiológico, com delineamento transversal, norteado pela ferramenta STROBE (*Strengthening the Reporting of Observational Studies in Epidemiology*) e desenvolvido a partir de dados do projeto intitulado “Parto e Aleitamento Materno em Filhos de Mães Infectadas por SARS-CoV-2”. A pesquisa nos prontuários das parturientes foi realizada no ano de 2020 em três maternidades referência na cidade de Belo Horizonte, Minas Gerais, Brasil, e, posteriormente, os contatos telefônicos foram realizados no período de 2020 a 2022 com as puérperas.

Um dos hospitais atende um quantitativo de usuários que suplanta o valor de 400 mil habitantes oriundos da cidade de Belo Horizonte e de outros municípios, mediante a Central Reguladora da Secretaria Municipal de Saúde de Belo Horizonte. Essa instituição trata-se de um hospital filantrópico localizado no Distrito Sanitário Norte, destacado pelo MS como modelo de assistência baseado em evidências científicas nas práticas humanizadas ao recém-nascido, bem como pela adoção da Rede Cegonha como política pública de saúde, perfazendo um total de 951 partos assistidos ao mês^(11)^.

O segundo hospital assiste mensalmente cerca de 250 partos, além de ser um local de relevância para as demandas de urgência e emergência e de saúde materno-infantil. É uma instituição filantrópica gerenciada pela Universidade Federal de Minas Gerais^(12)^. Por fim, o terceiro é um hospital público-regional, que oferece serviços de urgência, acolhimento integral a pacientes com condições respiratórias e que apresentem patologias complexas, assistência global à saúde da criança e do adolescente, dentre outros. No entanto, recebe notoriedade no âmbito da ginecologia e obstetrícia a partir da atenção ofertada à mulher^(13)^.

Referente ao cálculo amostral, apropriou-se do delineamento de estudo de coorte, sendo considerada uma razão de nove gestantes para o grupo controle (grávidas não expostas à COVID-19) para cada gestante do grupo caso (mulher exposta à COVID-19), em virtude da elevada taxa de infecção de 10% no período da epidemia^(14)^. Para obter um intervalo de confiança de 95% e poder de 80% para a amostra, estimou-se a *Odds Ratio* de 1,5. Portanto, fundamentado nesses parâmetros, resultou-se em uma amostra final composta por 2.267 parturientes, sendo que, para a divisão de gestantes por maternidade, considerou-se a proporção do número total de nascimentos de cada instituição definida. As gestantes foram acessadas em horários diversos, e o contato telefônico foi feito por pelo menos 5 tentativas por pesquisadores capacitados. Em caso de negativas ou insucesso nessas tentativas, a puérpera foi excluída/substituída. Assim, o seguimento da coleta de dados foi composto por 360 gestantes que responderam ao contato telefônico durante o seguimento da coleta de dados.

### Amostra, critérios de inclusão e exclusão

No que tange à seleção amostral, escolheu-se o período de maior incidência dos casos de COVID-19 para análise dos prontuários, sendo eles os meses de maio, junho e julho de 2020. Dentre esses prontuários, selecionaram-se aqueles que atendiam aos critérios de inclusão, sendo consideradas elegíveis, no montante total das análises, todas as grávidas de gestação única com parto hospitalar, que tiveram recém-nascidos (RN) concebidos com 22 semanas gestacionais ou mais; RN vivos e com peso acima de 500 gramas ao nascer; sendo descartadas mulheres com idade inferior a 18 anos. Finalmente, por seleção randômica, as parturientes foram escolhidas a partir do livro de registro e parto e, subsequentemente, seus prontuários foram avaliados nos hospitais de referência.

### Protocolo do estudo

A coleta dos dados ocorreu por profissionais capacitados a partir da análise dos prontuários selecionados de cada instituição hospitalar em estudo. Utilizou-se, como instrumento de coleta, um questionário estruturado adaptado da pesquisa “Parto e Aleitamento Materno em Filhos de Mães Infectadas por SARS-CoV-2” a fim de analisar a história pregressa clínica-obstétrica, a assistência ao trabalho de parto e parto nas instituições observadas, as vias de nascimento, as alterações clínicas maternas durante toda a internação, o aleitamento materno e a infecção por COVID-19. A constatação ou não de infecção da mulher por SARS-CoV-2 foi verificada a partir dos prontuários hospitalares, sendo que, em mulheres sintomáticas, buscou-se o teste confirmatório efetuado na instituição e seu respectivo resultado. No caso de ausência da realização do teste, julgou-se como suspeitas as gestantes que apresentavam sintomas sugestivos da infecção no momento da admissão.

A variável dependente foi a não vacinação contra a COVID-19 em gestantes de Belo Horizonte. As variáveis independentes foram divididas em variáveis sociodemográficas (idade, escolaridade, renda, situação conjugal, cor de pele e ocupação durante a gestação), relacionadas ao histórico obstétrico (paridade, histórico de aborto e número de consultas de pré-natal), relacionadas a micropolíticas (acesso dificultado a consultas de pré-natal e recebimento de orientações contra COVID-19 durante o pré-natal) e relacionadas a macropolíticas (recebimento da vacina contra COVID-19).

### Análise dos resultados e estatística

Os dados obtidos foram armazenados em uma planilha eletrônica no programa *Microsoft Office Excel*® 2010. Em seguida, foram analisados por meio do pacote estatístico *Statistical Software for Professional* (Stata), versão 17.0, e apresentados por meio de frequência absoluta e relativas. As variáveis foram controladas por idade, paridade, histórico de aborto, escolaridade, número de consultas de PN, renda, situação conjugal e cor de pele autor-referida. Inicialmente, os dados categóricos, foram apresentados por meio de frequência absoluta, relativa e seus respectivos intervalos de confiança (IC 95%). Realizou-se, também, a regressão de Poisson para estimar os parâmetros de interesse: brutos e ajustados dos fatores associados a não vacinação de COVID-19 em puérperas de Belo Horizonte. A construção do modelo de regressão multivariado seguiu o método backward; e incluíram-se todas as variáveis de interesse em nível de significância estatística inferior a 20% na análise bivariada e ou critério teórico^(15)^. O teste de Hosmer-Lemeshow foi utilizado para verificar o ajuste do modelo final. As razões de prevalências brutas e ajustadas foram apresentadas e os intervalos 95% de confiança (IC95%) calculados, considerando-se um nível de significância de 5% em todos os procedimentos analíticos.

## RESULTADOS

A amostra deste estudo foi composta por 360 gestantes e, destas, 77,89% receberam a vacina contra a COVID-19. Em relação ao perfil sociodemográfico, 59,36% das gestantes tinham idade inferior a 30 anos; 73,06% possuíam ensino superior ou ensino médio completo; 50% tinham renda de até 1 salário-mínimo ou mais ou então não possuíam renda; 60,83% eram casadas ou apresentavam união estável; 84,44% disseram ter cor autorreferida preta, parda, amarela ou indígena; além disso, 66,22% afirmaram trabalhar durante a gestação.

No que se refere ao histórico obstétrico, 88,61% eram multíparas; 72,76% não apresentavam histórico de aborto; 84,05% referiram número de consultas de pré-natal superior a 6; 67,78% afirmaram não ter dificuldade no acesso às consultas de pré-natal e 88,06% tiveram rede de apoio pós-parto. Por fim, em relação à vacinação contra a COVID-19, 65,03% receberam orientações quanto à COVID-19; 77,89% delas foram vacinadas, sendo que 45,92% se imunizaram com a vacina Pfizer (Tabela 1).

**Tabela 1:** Perfil demográfico, socioeconômico e obstétrico da amostra de gestantes. Belo Horizonte, Minas Gerais, Brasil, 2020-2022 (N=360)

	n (%)	IC95%
Perfil sociodemográfico		
Idade		
Menor ou igual a 30 anos	149 (59,36)	53,12-65,30
Maior que 30 anos	102 (40,64)	34,69-46,87
Escolaridade		
Superior/Ensino médio	263 (73,06)	68,21 -77,40
Fundamental/Primário/Analfabeta	97 (26,94)	22,59 – 31,78
Renda		
1 salário-mínimo ou mais	174 (50,0)	44,74-55,25
Sem renda/até 1 salário-mínimo	174 (50,0)	44,74-55,25
Situação Conjugal		
Casada/união estável	219 (60,83)	55,66-65,76
Solteira/viúva/divorciada	141 (39,17)	34,23-44,33
Cor de pele		
Branca	56 (15,56)	12,15-19,69
Preta/Parda/amarela/indígena	304 (84,44)	80,30-87,84
Trabalhava durante a gestação		
Sim	224 (66,22)	57,07-67,10
Não	136 (37,78)	32,89-42,92
Histórico Obstétrico		
Paridade		
Primípara	41 (11,39)	08,48-15,12
Multípara	319 (88,61)	84,87-91,51
Histórico de aborto		
Não	235 (72,76)	67,61-77,35
Sim	88 (27,24)	22,64-32,38
Número de consultas de PN		
Maior ou igual a 6	216 (84,05)	79,01- 88,05
Menor que 6	41 (15,95)	11,94-20,98
Acesso Consulta PN dificultado		
Não	244 (67,78)	62,74-72,42
Sim	116 (32,22)	27,57-37,25
Rede de apoio pós-parto		
Sim	317 (88,06)	84,25-91,03
Não	43 (11,94)	08,96-15,74
Vacinação		
Receber orientações contra COVID-19 PN		
Sim	186 (65,03)	59,29-70,37
Não	100 (34,97)	29,62-40,70
Recebeu a vacina contra COVID-19		
Sim	236 (77,89)	72,83-82,22
Não	67 (22,11)	17,77-27,16
Qual vacina?		
CoronaVac	61 (26,18)	20,90-32,24
Oxford-Astrazeneza	63 (27,04)	21,68-33,14
Pfizer	107 (45,92)	39,57-52,40
Janssen	02 (00,86)	00,21-03,39

*Notas: n= Número amostral; IC95% = Intervalo de confiança de 95%; Os totais das variáveis (n) podem variar devido à perda de dados em cada uma delas.*

Por meio da análise da razão de prevalência e da análise bivariada dos fatores associados à não vacinação contra a COVID-19 entre as gestantes do estudo, observou-se que predominou, entre as gestantes não vacinadas: idade inferior a 30 anos (68,39%), multíparas (22,47%), ensino fundamental/primário ou analfabetas (25,86%), ausência de histórico de aborto (24,10%), número de consultas de pré-natal acima de 6 (18,09%), relato de não recebimento de orientações quanto à COVID-19 no pré-natal (20,69%), ausência de renda ou renda até 1 salário mínimo (27,34%), estado civil solteira/ viúva/divorciada (25,86%), cor de pele autorreferida preta/parda/ amarela/indígena (22,44%), não trabalhavam durante a gestação (29,31%), tiveram dificuldade de acesso às consultas de pré-natal (23,71%) e relato de não possuírem rede de apoio (38,24%).

Observou-se diferença estatisticamente significativa (p<0,018) em relação ao fato de a mulher trabalhar durante a pandemia, bem como no que tange à rede de apoio (p<0,010). Na análise bivariada, observou-se associação entre a não vacinação e o trabalho remunerado durante o pré-natal e rede de apoio, evidenciando-se diferença estatisticamente significativa (Tabela 2).

**Tabela 2:** Análises de prevalência de não vacinação e análise bivariada dos fatores associados à não vacinação contra a COVID-19 em gestantes. Belo Horizonte, Minas Gerais, Brasil, 2020-2022

	Não vacinadas n=67	Análise Bruta	
	n (%)	RP^1^(IC95%)	Valor de *p*
Idade			0,188
Menor ou igual a 30 anos	31 (68,39)		
Maior que 30 anos	14 (31,11)	0,682 (0,387-1,204)	
Paridade			0,686
Primípara	7 (19,44)		
Multípara	60 (22,47)	1,151 (0,572 – 2,332)	
Escolaridade			0,777
Superior/Ensino médio	37 (19,79)	1	
Fundamental/primário/analfabeta	30 (25,86)	0,931 (0,571-1,519)	
Histórico de aborto			0,226
Não	47 (24,10)	1	
Sim	13 (17,11)	0,709 (0,407-1,236)	
Número de consultas PN			0,895
Maior ou igual a 6	34 (18,09)	1	
Menor que 6	6 (17,14)	0,947 (0,429-2,090)	
Receber orientações COVID-19 PN			0,955
Sim	32 (20,38)	1	
Não	18 (20,69)	1,012 (0,667-1,534)	
Renda			0,056
1 salário-mínimo ou mais	28 (17,95)	1	
Sem renda/até 1 salário-mínimo	38 (27,34)	1,523 (0,998-2,346)	
Situação conjugal			0,215
Casada/união estável	37 (19,79)	1	
Solteira/viúva/divorciada	30 (25,86)	1,307 (0,856-1,995)	
Cor de pele			0,881
Branca	10 (21,28)	1	
Preta/Parda/Amarela/Indígena	57 (22,44)	1,04 (0,899-1,131)	
Trabalhava durante a gestação			0,018
Sim	33 (17,65)	1	
Não	34 (29,31)	1,660 (1,091-1,527)	
Acesso consultas PN dificultados			0,644
Não	44 (21,36)	1	
Sim	23 (23,71)	1,110 (0,712-1,729)	
Rede de apoio			0,010
Sim	54 (20,07)	1	
Não	13 (38,24)	1,904 (1,166-3,109)	

*Notas: n= Número amostral; RP = Razão de prevalência; IC95% = Intervalo de confiança de 95%; Os totais das variáveis (n) podem variar devido à perda de dados em cada uma delas.*

Nas análises ajustadas, observou-se que, após ajuste para as demais variáveis, a condição de não trabalhar durante a gestação aumentou, em média, 2,15 vezes a razão de prevalência de a mulher não receber a vacina contra a COVID-19 em comparação com aquelas que trabalhavam durante a gestação. Ter dificuldade em acessar serviços de saúde durante o pré-natal aumentou, em média, 1,93 vezes a razão de prevalência de a mulher não receber a vacina contra a COVID-19, quando comparadas às mulheres que não tiveram dificuldades de acessar o serviço de saúde. Não ter rede de apoio aumentou, em média, 2,47 vezes a razão de prevalência de a mulher não receber a vacina contra a COVID-19, quando comparadas àquelas que tinham algum tipo de rede de apoio no puerpério (Tabela 3).

**Tabela 3:** Análises de prevalência de não vacinados e análises bivariadas dos fatores associados à não vacinação contra a COVID-19 em gestantes. Belo Horizonte, Minas Gerais, Brasil, 2020-2022

	Análise Ajustada^2^	
	RP² (IC95%)	Valor de *p*
Trabalhava durante a gestação		
Sim	1	
Não	2,15 (1,759-3,957)	0,013
Acesso consultas PN dificultados		
Não	1	
Sim	1,93 (1,044-3,576)	0,036
Rede de apoio		
Sim	1	
Não	2,47 (1,322-4,621)	0,005

*Notas: RP = Razão de prevalência; IC95% = Intervalo de confiança de 95%.*

## DISCUSSÃO

Neste trabalho, observou-se que 77,89% das gestantes foram vacinadas contra a COVID-19, revelando que uma parcela significativa deste grupo não aderiu à vacinação. Além dos fatores sociodemográficos e do histórico obstétrico analisados, outros fatores externos ou sociais, como os inerentes aos serviços de saúde ou à rede de apoio, além de trabalhar ou não durante a gestação, podem influenciar as puérperas a não se vacinarem. Um estudo desenvolvido no Paquistão em 2020 relatou que existem fatores, como características demográficas e financeiras, que interferem no processo de hesitação vacinal contra a COVID-19^(16)^.

No que se refere à cobertura vacinal, existe uma relação importante entre as desigualdades obstétricas e a vacinação. O reconhecimento da determinação social da saúde pressupõe considerar questões sociais, econômicas, culturais e ambientais que atuam sobre a saúde do indivíduo, influenciando as desigualdades sociais existentes e, consequentemente, a redução das coberturas vacinais em determinados grupos populacionais^(17)^.

As condições desiguais em que se encontram certas pessoas correspondem às iniquidades em saúde, as quais se referem a determinados contextos em que há acesso desigual, ou até mesmo falta de acesso aos direitos humanos fundamentais que garantem um nível mínimo necessário para proporcionar uma vida digna e justa^(18)^.

Um estudo realizado na Itália entre 2005 e 2010, com dados obtidos da fonte administrativa da Certidão Padrão de Nascidos Vivos, demonstrou uma relação de dependência entre o uso de serviços de pré-natal (PN) e os aspectos sociodemográficos^(19)^. Sabe-se que mulheres com baixa escolaridade, solteiras e desempregadas possuem uma chance maior de se apropriarem inadequadamente dos serviços de pré-natal. Da mesma forma, contextos familiares “disfuncionais”, como pais solteiros ou relacionamento não saudável entre a figura paterna e a criança, interferem consideravelmente nas consultas de pré-natal^(19)^.

No presente estudo, as mulheres sem companheiro apresentaram menor acesso às consultas de pré-natal e início mais tardio da assistência. Em contraposição, existe uma forte associação entre estado civil e uso adequado de pré-natal, à medida que mulheres casadas ou que vivem com seu companheiro têm maior facilidade em acessar o serviço, já que contam com rede de apoio para auxiliá-las neste processo, inclusive nas ações de imunização^(20)^.

Um estudo recente com mulheres grávidas na Suécia evidenciou que muitas delas foram privadas de informações dos profissionais de saúde durante o período da pandemia, incluindo também as ações de imunização^(21)^. Um estudo transversal australiano afirmou que somente um terço das gestantes pesquisadas recebeu educação pré-natal de qualidade^(21)^.

A adequada assistência de pré-natal é um instrumento imprescindível para a promoção da saúde da mulher no período gravídico-puerperal, sendo que as práticas realizadas durante esse processo estão associadas a melhores desfechos perinatais. Diante disso, o profissional de saúde, ao estabelecer um vínculo com a gestante, consegue orientá-la sobre os cuidados na gestação, como, por exemplo, a importância da vacinação contra a COVID-19 para sua proteção e do seu bebê.

Verificou-se que, neste estudo, o fator socioeconômico e o acesso aos serviços de saúde demonstram ser determinantes para a maior cobertura vacinal no período gestacional. Foi identificado, ainda, que o trabalho remunerado e o número de consultas de pré-natal associaram-se a uma menor proporção de ausência de vacinação nas gestantes. Portanto, o vínculo empregatício caracteriza-se como fator importante para a adequação do pré-natal, uma vez que estudos semelhantes constatam uma conexão entre a mulher trabalhar e o início precoce das consultas de pré-natal, o que gera, por conseguinte, uma maior probabilidade de imunização materna contra a COVID-19 e outras doenças^(22)^.

Logo, o fato de a mulher trabalhar pode influenciar positivamente no acesso ao pré-natal e nos cuidados com sua saúde, em virtude do maior acesso à informação, bem como da relação com um nível elevado de escolaridade. A assistência prestada durante o período de pré-natal está diretamente relacionada à vacinação de gestantes, sendo um fator significativo que afeta diretamente a cobertura vacinal, já que proporciona um conhecimento mais significativo da gestante acerca da proteção conferida pelos imunobiológicos^(22)^.

As gestantes empregadas (79,4%) apresentaram um nível de aceitação da vacina contra a COVID-19 2,44 vezes maior do que as gestantes que não trabalhavam (52%). Ademais, os empregadores podem estimular ou mesmo determinar como exigência a vacinação como parte de suas condições de trabalho, sobretudo em alguns setores, e, desse modo, as gestantes empregadas poderiam ter uma adesão maior à vacinação^(23)^. As mães empregadas podem usar os serviços de pré-natal com mais frequência porque as informações sobre os riscos da gravidez estão amplamente disponíveis no local de trabalho^(24)^.

### Limitações do Estudo

Por fim, salientam-se algumas limitações deste trabalho, como o fato de, entre as 67 gestantes não vacinadas, não encontrarmos outras associações para a não vacinação. Ou seja, há a possibilidade de existirem fatores adicionais a serem investigados, além da vontade individual ou do desejo real de não se vacinar. Todavia, reforça-se o uso de uma metodologia rigorosa na execução deste estudo e a escassez de trabalhos nacionais acerca dessa temática, para o nosso conhecimento.

### Contribuições para a Área da Enfermagem

O presente estudo contribui para a área da Enfermagem à medida que apresenta informações que podem nortear estudos futuros sobre a temática, direcionando os profissionais da equipe multidisciplinar no seu cotidiano e influindo positivamente na prática desses profissionais. Ademais, pode ser utilizado como fonte de informação para educação e orientação em saúde realizada pela equipe de enfermagem ao público-alvo, contribuindo de forma positiva e aditiva nos dados relacionados aos desfechos obstétricos da SARS-CoV-2 em puérperas vacinadas e não vacinadas. Além disso, pode contribuir para o aumento da cobertura vacinal, tornando a vacina mais acessível devido às informações relevantes.

## CONCLUSÕES

A partir deste trabalho, conclui-se que determinantes externos, sociais e institucionais podem influenciar na menor adesão à vacinação contra a COVID-19, em especial: ausência de trabalho durante a gestação, acesso dificultado a consultas de pré-natal, além de rede de apoio comprometida ou deficiente. Diante dos achados aqui encontrados, este estudo reforça e avança na compreensão sobre os benefícios da vacinação contra a COVID-19 para a população de mulheres.

Diante desse cenário, é necessário que haja um maior incentivo à educação em saúde, sobretudo no momento da assistência ao pré-natal, a fim de que o profissional de saúde reforce a relevância da vacinação na gestação e no pós-parto como fator protetivo, resultando em menores taxas de morbimortalidade e, por consequência, em desfechos perinatais favoráveis. Espera-se que este estudo possa contribuir para a adesão das puérperas à vacinação contra a COVID-19, visando mitigar os efeitos negativos da pandemia mediante a prevenção de doenças, como o coronavírus.
